# Interrupted Blood Feeding in Ticks: Causes and Consequences

**DOI:** 10.3390/microorganisms8060910

**Published:** 2020-06-16

**Authors:** Djamel Tahir, Leon Meyer, Josephus Fourie, Frans Jongejan, Thomas Mather, Valérie Choumet, Byron Blagburn, Reinhard K. Straubinger, Marie Varloud

**Affiliations:** 1 Clinvet Morocco, B.P 301, Mohammedia 28815, Morocco; djamel.tahir@yahoo.fr (D.T.); Leon.Meyer@clinvet.com (L.M.); 2 Clinvet International, P.O. Box 11186, Universitas, Bloemfontein 9321, South Africa; josephus.fourie@clinvet.com; 3 Utrecht Centre for Tick-Borne Diseases, FAO Reference Centre for Ticks and Tick-Borne Diseases, Faculty of Veterinary Medicine, Utrecht University, Yalelaan 1, 3584 CL Utrecht, The Netherlands; FJongejan@TBDInternationalbv.com; 4 Center for Vector-Borne Disease, University of Rhode Island, Kingston, RI 02881, USA; tmather@uri.edu; 5 Environnement et Risques Infectieux, Institut Pasteur, 75015 Paris, France; valerie.choumet@pasteur.fr; 6 College of Veterinary Medicine, Auburn University, Auburn, AL 36849, USA; blagbbl@auburn.edu; 7 Institute for Infectious Diseases and Zoonoses, Bacteriology and Mycology, Ludwig-Maximilians-University Munich, 80539 Munich, Germany; Reinhard.Straubinger@micro.vetmed.uni-muenchen.de; 8 Ceva Santé Animale, 10 Avenue de la Ballastière, 33500 Libourne, France

**Keywords:** interrupted blood feeding, ixodid ticks, transmission time, pathogens

## Abstract

Ticks are obligate hematophagous arthropods and act as vectors for a great variety of pathogens, including viruses, bacteria, protozoa, and helminths. Some tick-borne viruses, such as Powassan virus and tick-borne encephalitis virus, are transmissible within 15–60 min after tick attachment. However, a minimum of 3–24 h of tick attachment is necessary to effectively transmit bacterial agents such as *Ehrlichia* spp., *Anaplasma* spp., and *Rickettsia* spp. to a new host. Longer transmission periods were reported for *Borrelia* spp. and protozoans such as *Babesia* spp., which require a minimum duration of 24–48 h of tick attachment for maturation and migration of the pathogen. Laboratory observations indicate that the probability of transmission of tick-borne pathogens increases with the duration an infected tick is allowed to remain attached to the host. However, the transmission time may be shortened when partially fed infected ticks detach from their initial host and reattach to a new host, on which they complete their engorgement. For example, early transmission of tick-borne pathogens (e.g., *Rickettsia rickettsii*, *Borrelia burgdorferi*, and *Babesia canis*) and a significantly shorter transmission time were demonstrated in laboratory experiments by interrupted blood feeding. The relevance of such situations under field conditions remains poorly documented. In this review, we explore parameters of, and causes leading to, spontaneous interrupted feeding in nature, as well as the effects of this behavior on the minimum time required for transmission of tick-borne pathogens.

## 1. Introduction

Ticks are obligate hematophagous Acari that parasitize vertebrate animals and occasionally bite humans [1]. They play a role as vectors and/or reservoirs for a variety of pathogenic microorganisms (e.g., bacteria, protozoa, viruses, and helminths). Ticks become infected with these microorganisms by feeding on infective hosts (human or animal) and, after a cycle of biological development, later inject the microorganisms into the new host during their subsequent blood meal [2,3]. In some, but certainly not all cases, female ticks also can transmit pathogens to their eggs; in such cases, newly hatched larvae will already be infected [4]. Furthermore, ticks are considered to be the second most common vector of pathogens, after mosquitoes, for human infectious diseases [1,5,6]. They also play a primary role as vectors of pathogens for animals, resulting in a multitude of infectious diseases worldwide [7].

There are three families of ticks, two of which are considered to be of veterinary and medical significance—*Ixodidae* (hard ticks) and *Argasidae* (soft ticks), which are composed of 702 and 193 described species, respectively [8,9]. The family Nuttalliellidae is represented by the monospecific genus *Nuttalliella*, which comprises a single species—*Nuttalliella namaqua* [10]. Most of the approximately 100 human and animal arthropod-borne infections are associated with just 116 tick species [7]. The vectorial capacity of ixodid ticks is related to their long-term co-evolution with the pathogens that they transmit, their longevity, their high reproductive potential, the wide range of host feeding preferences of several species, and their capacity to imbibe a substantial quantity of blood over a relatively long period of time [3,11].

Ixodid ticks have four developmental stages—egg, larva, nymph, and adult. After hatching from the eggs, ticks need to feed on hosts at least once during each stage. They are pool feeders that inject saliva during feeding and complete engorgement in an alternating pattern of salivating and sucking through the same canal. As such, salivary glands play an important role in tick feeding and pathogen transmission [11,12,13]. During feeding, hard ticks remain attached to the host for a few days or up to two weeks depending on the species and stage of development [14]. Some hard ticks cement their mouthparts into the skin early on during the attachment process and will only release them during the rapid feeding phase (the final 12–36 h) when most of the body fluid and erythrocyte meal has been taken up [14]. Importantly, the tick-borne pathogens that ticks transmit frequently require a period of reactivation and/or replication prior to being transmitted, via saliva, from an infected tick to a naive host [15]. For example, the efficient transmission of pathogens by ixodid ticks requires in general a minimum time of 15 min of tick attachment for powassan virus [15], 1 h of tick attachment for tick-borne encephalitis virus [16], 3–24 h of tick attachment for *Ehrlichia* spp. [17], *Anaplasma* spp. [18], and *Rickettsia* spp. [19], and 24–48 h of tick attachment for *Babesia* spp. [20] and *Borrelia* spp. [21,22]. These variations in the transmission time can be explained by the presence of pathogens in salivary glands of unfed ticks at the time of blood feeding as demonstrated in anterior studies on *Anaplasma* spp., *Ehrlichia* spp., and *Rickettsia* spp. [23,24]. In contrast, *Borrelia* spp. are known to be restricted to the midgut of *Ixodes scapularis* ticks and migrate to the salivary glands during the blood meal [25]. All *Borrelia burgdorferi* s.l. genospecies except *Borrelia afzelii* have been detected in both the midgut and salivary glands of *Ixodes ricinus* ticks [24].

Kahl et al., in their study [26] conducted on *I. ricinus* nymphs infected with *B. burgdorferi* sensu lato originating from field-collected ticks, found that four of six (67%) and three of six (50%) of the experimental hosts (gerbils) had acquired a transmissible infection after 16.7 and 28.9 h, respectively, of tick feeding. A recent study demonstrated that *Borrelia*-infected *I. ricinus* nymphs were able to infect mice after just 12 h of tick attachment [27], which represents the shortest duration of attachment reported for *Borrelia burgdorferi* s.l. transmission. However, several studies report that larvae, nymphs, and adult ticks whose blood feeding has been interrupted and who have access to a new host will readily reattach and resume feeding [19,28,29,30,31,32]. Termed “interrupted feeding”, the ability of partially fed ticks to survive, reattach to a new host, and complete engorgement is supported by their ability to switch their salivary gland gene expression pattern as demonstrated in *Rhipicephalus appendiculatus* ticks [33]. This interrupted feeding pattern, in which ticks continue to feed on a second host organism during the same feeding cycle, was demonstrated to result in reduced transmission times for the *B. burgdorferi*, *Rickettsia rickettsii*, and *Babesia canis* tick-borne pathogens (Table 1).

Herein, we (i) review the evidence for interrupted feeding, obtained either experimentally or naturally, in ticks; (ii) investigate the causes of interrupted feeding in ticks; and (iii) discuss the effects of interrupted feeding on the minimum times required for transmission of tick-borne pathogens.

## 2. Evidence for Interrupted Feeding in Ticks

Several direct observations of interrupted tick feeding have been made under both controlled and natural conditions [34]. The aptitude of partially engorged ticks to reattach and continue feeding was also documented by Varma et al. [35]. These authors reported the successful reattachment of *Haemaphysalis spinigera* nymphs to new animal hosts, on which they went on to feed to repletion. Similarly, subsequent studies showed that *Hyalomma isaaci* nymphs that had partially engorged on a different initial host [36], and *Ixodes persulcatus* larvae and nymphs [37], successfully completed engorgement on a second host. However, the latter authors found, even in ticks that had been transferred to a new host and fed to repletion, that successful development to the next stage did not always occur in larvae and nymphs that experienced an interrupted feeding cycle [37]. When laboratory mice were infested with *I. persulcatus* ticks and euthanized 24 or 48 h after exposure, larvae and nymphs were found, starting 3 h after euthanasia, to spontaneously detach from the dead hosts. The same ticks subsequently continued feeding on new hosts in order to complete engorgement [30]. Fifty-seven percent of the larvae and 80% of the nymphs succeeded in feeding to repletion [30]. Similar findings were reported for *Ixodes scapularis* larvae using hamsters as hosts. Animals with larvae attached to them were sacrificed at specified time intervals. The larvae subsequently detached from the dead animals over the next two days following the host’s death. After 18 h of attachment to an initial host and five weeks of tick incubation, semi-engorged *I. scapularis* larvae were deposited onto a new host. Thirty-eight percent (19/50) of the larvae reattached to the new host and fed to repletion [28]. To date, no data have been obtained to demonstrate that *I. scapularis* nymphs naturally detach following the host’s death. However, it has been reported that semi-engorged nymphs were observed to detach spontaneously from free-ranging mice in the laboratory, perhaps as frequently as 15% of the time [31]. In addition, partially engorged *I. scapularis* nymphs successfully reattached to a second host and fed to repletion after spending up to 48 h on the initial host [31]. Interestingly, partially engorged *I. ricinus* nymphs have occasionally been observed to search for a new host in nature [38]. Taken together, these observations suggest that in nature, regardless of the tick species, undamaged ticks that have not imbibed the amount of blood necessary for molting are capable of reattaching to another host to complete their blood feeding [33].

Ticks may prematurely detach from their hosts for various reasons, including scratching or vigorous shaking of the host, the host’s immune response, or the host’s death. A large number of partially engorged ixodid ticks were found on the forest floor soon after the death of heavily tick-infested monkeys infected with Kyasanur Forest Disease (KFD) virus. These tick larvae and nymphs were collected and subsequently deposited on laboratory animals (monkeys, squirrels, and mice), on which they reattached and successfully completed their blood meal [29]. The results obtained by Wang et al. [39] showed that when hamsters infected with Thogoto virus were used as hosts, all partially fed nymphal and adult *R. appendiculatus* ticks detached themselves within 3 days following the viremia-induced death of the hamsters. In this case, all of the partially fed nymphs (60/60) survived the 14-day feeding interruption and successfully reattached and fed to repletion when placed on new, uninfected hosts [39].

For most ixodid ticks, males are naturally intermittent feeders. The ixodid ticks are classified morphologically as either Metastriata (several genera) or Prostriata (only *Ixodes* spp.). While spermatogenesis occurs prior to adult feeding in Prostriata ticks, males of most of the species in the Metastriata group require attachment to a host and blood feeding to initiate sperm development [14]. However, once stimulated, these males can readily detach from their hosts and move about in search of a female tick to mate with. Male *Dermacentor andersoni* ticks were observed to move between cattle in 48% (26/54) of the animal pairs [40]. Field observations reported for *Dermacentor reticulatus* ticks suggest that this behavior occurs under natural conditions for this tick species as well. Although adult *D. reticulatus* ticks are characterized by aggressiveness, questing adult ticks were found at a distance of 23 m from their original location. This distance is too great for their locomotor activity, which varies around 60 cm, suggesting on-host transfer with subsequent detachment under natural conditions. This movement of adult ticks increases as the humidity in their habitat increases [41]. Interrupted feeding and spontaneous tick migration between dog hosts was also reported to occur for *Rhipicephalus sanguineus sensu lato* ticks [42]. After attachment to an initial dog host, ticks were observed to move two days after infestation, with 13.3–46% of the *R. sanguineus* ticks that were present on the initial dog hosts transferring to other co-housed dogs. In a more recent study, two authors of this manuscript [32] demonstrated the ability of adult *D. reticulatus* males to reattach to a new host after having been attached to an initial host (sheep or dog) for 72 and 88 h, with a mean rate of reattachment to the second host (dog) of 58% (29/50) and 47% (23/50), respectively. In such situations, where female-seeking male ticks move between hosts, it is possible that they acquire pathogens from infected former hosts. When ticks move to another host upon whom they resume feeding, they accomplish the (possibly accelerated) transmission of disease agents [43].

Other indirect evidence of interrupted feeding in ticks includes detection of DNA from different host species in ticks and the detection of pathogens in hosts at life stages during which they are not expected to be infected. DNA from different hosts in questing ticks has been detected in several studies conducted in Europe and North America. Partially engorged larvae of *I. ricinus* were occasionally collected by flagging in the Killarney National Park, Ireland. A determination of the content of a blood meal revealed the presence of DNA from more than one host species, suggesting that these ticks had ingested blood from multiple hosts [44]. Similar observations were reported in Chaumont Mountain, Switzerland, where DNA from multiple vertebrate hosts was detected in 19.5% (71/364) and 18.9% (40/214) of nymphs and adults, respectively, of *I. ricinus* [45]. In the Neuchâtel area, Switzerland, the same study reported the presence of a mixture of vertebrate host DNA in 5.6% (3/53) of *I. ricinus* ticks, involving 9.5% (2/21) of the nymphs and 3.2% (1/32) of the adults [46]. Similar results were reported in a study conducted in forest patches in the province of Trento, Italy, where 10.7% (23/215) of collected *I. ricinus* nymphs contained mixed blood meals [47]. In the Czech Republic, the DNA of two different types of vertebrate hosts was successfully detected in 15.8% (42/266) of *I. ricinus* nymphs [48]. Regarding *I. scapularis*, a study conducted in the United States reported that, of a total of 79 adult ticks collected by dragging vegetation in deciduous forests in Tennessee, Rhode Island, and Wisconsin, the blood meal sources contained the DNA of two host vertebrate species in four samples (9.5%) out of a total of 42 [49]. Regarding other ixodid tick species, the DNA of multiple vertebrate species was detected in 16.2% (141/869) of *Amblyomma americanum* nymphal ticks collected in St. Louis, MO, USA [50]. Scott et al. reported that 23.7% (14/59) of *A. americanum* nymphs collected in TN, USA contained mixed blood meals. These observations are supported by a subsequent study in which mixed blood meal sources were identified in almost 9% (12/137) and 20% (10/52) of questing *A. americanum* adult and nymphal ticks, respectively, collected from vegetation at the Cumberland Plateau retirement community, USA [51]. These DNA determination techniques likely underestimate the occurrence of interrupted feeding, since they cannot detect the multiple feedings that likely occur on different individuals of the same host species or genera.

Due to the reported lack of transovarial transmission of *Borrelia burgdorferi*, detection of spirochetes would not be expected in apparently unfed larval stages of *Ixodes* spp. ticks [28,52,53,54]. However, *B. burgdorferi* s.l.-infected *I. ricinus* larvae, questing and apparently unfed, have been reported in multiple borreliosis surveillance studies (Table 2). For all of these investigations, it remains undetermined whether the larvae acquired their spirochete infection from a host during an interrupted blood meal, or whether transovarial transmission of *B. burgdorferi* may only rarely occur. Nevertheless, it seems that even a brief interruption in feeding on an infected host may be sufficient for a larva to acquire a pathogen, including spirochetes [55]. In fact, it has been shown that a blood meal that lasts for 4 and 8 h is sufficient for the majority of *I. ricinus* larvae to become infected with *B. afzelii* and *B. burgdorferi* s.s, respectively [55]. This suggests that the detection of a spirochetal infection in questing larvae might be explained by an interrupted blood meal as demonstrated in a study in which partially engorged ixodid ticks failed to develop to the next stage [37].

Accordingly, the mixed blood meals that were detected in ticks at the larval, nymphal, and adult life stages, as well as the pathogens that were detected in unfed larvae of *I. ricinus* collected in nature (Table 3), could be due to interrupted blood feeding, in which individual ticks are capable of feeding on more than one host. This hypothesis is supported by the fact that ticks can, for various reasons, detach from one host and successfully reattach to another host.

## 3. How Do Ticks Manage Interrupted Blood Meals?

Ixodid ticks commonly require a single bloodmeal to transition from one life stage to another [14]. In the three-host life cycle, which is the most common developmental pattern, ixodid ticks will, at each active stage, seek a host, feed, and then drop off to develop further in appropriate niches in the natural environment [3]. However, as discussed above, ixodid ticks can also detach from a host before repletion and complete their interrupted blood meal on a different host.

The death of the host and the cooling of its body can cause ticks to spontaneously detach [66]. In fact, many blood-sucking parasites, including fleas, ticks, and lice, require a certain body surface temperature in order to attach to, remain attached to, and feed on their hosts. Some tick species (e.g., *Amblyomma rotundatum, Ixodes pacificus*, and *I. scapularis*) are exceptions to this requirement and can feed on poikilothermic animals, such as snakes and lizards [67,68,69]. Additionally, the stoppage of blood flow in the capillaries may trigger the detachment of ticks from the lifeless host [29,30]. For example, *I. persulcatus* larvae and nymphs were found to begin detaching from dead mice as soon as 3 h after the host was euthanized, and no ticks were found to be attached to the carcasses after 21 h [30].

Several studies report that tick feeding induces host immune regulatory and effector pathways involving keratinocytes, natural killer cells, dendritic cells, T cell subpopulations, B cells, neutrophils, mast cells, basophils, endothelial cells, cytokines, chemokines, and complement [70,71,72,73]. Such immune interactions confer what is termed “acquired tick resistance” to hosts, resulting in a decreased tick blood-meal volume, a reduced tick engorgement weight, a prolonged feeding duration, reduced fecundity, and/or inhibition of molting [74]. For example, BALB/c mice developed resistance to *Dermacentor variabilis* larvae; this resistance was manifested by a reduction in the number of engorged larvae and in the weight of partially fed larvae. Such host resistance has mostly been noted during the third and fourth infestations [75]. In this situation, if partially engorged ticks detach from a tick-immune host, they are able to reattach to another host and take up the amount of blood necessary for molting [75].

Grooming and/or scratching behavior by the host has been shown to be effective in removing ectoparasites, including ticks [76,77,78]. Mooring and Samuel report that peak grooming rates in mice occurred during adult tick engorgement and depended on the degree of irritation caused by ticks during each of their life stages [76]. An engorging adult female tick appears to produce far more irritation than an engorging nymphal or larval tick. It was shown that increased self-grooming in white-footed mice (*Peromyscus leucopus*) reduced tick infestation burdens through the removal of immature feeding ticks. Once removed from their host, these immature ticks may seek another host if they have only obtained a partial blood meal [79]. Consequently, the possibility exists in nature of such infected ticks being dislodged by mechanical means (grooming and/or scratching), subsequently reattaching to another susceptible host, and then transmitting the infection during feeding to repletion [35]. However, it is worth noting that mechanical removal can cause a tick’s mouthparts to break off [80] and, consequently, inhibit the ability of the tick to reattach and successfully complete its blood meal.

As mentioned above, mate seeking is another means by which an interruption in blood feeding can occur among ixodid ticks, especially among Metastriate genera such as *Rhipicephalus* spp., *Amblyomma* spp., and *Dermacentor* spp. [3,81]. After attachment to and blood feeding on a host, which initiates sperm development, male metastriate ticks are able to detach to seek a female tick, and may even detach to seek another female after mating once or several times [43,82]. In their assessment of interrupted feeding and interhost migration of *R. sanguineus* ticks using co-housed dogs, male ticks were found to be especially motivated to seek a new host when females were not present on the same host [42]. For example, an average of 58.9% of male ticks originally on dog A (without female ticks) moved to dog B (with female ticks) [42]. It was observed that, before migrating between dogs, ticks were attached and presumably partially fed on the first dog for at least 48 h prior to changing hosts. This example could serve as a natural model for testing whether or not interrupted blood feeding might reduce the duration of attachment and accelerate the transmission of tick-borne pathogens [42].

After becoming detached when blood feeding has not been completed, some ticks have reportedly become more aggressive, perhaps losing or attenuating some of their discriminatory senses. It may be that partially fed ticks are programmed to complete their blood meal on any available vertebrate animal [83]. However, the cause of increased host seeking by partially fed ticks may not always be their blood feeding status. Belova et al. found partially engorged but tick-borne encephalitis virus (TBEV)-infected *I. ricinus* ticks more often on humans and animals than in nature, and suggested that the TBEV infection had changed the ticks’ behavior toward increased activity and aggressiveness [84].

## 4. Interrupted Blood Feeding and Its Effects on Transmission Times for Tick-Borne Pathogens

An interruption in an arthropod vector’s blood feeding increases the potential for mechanical transmission of pathogens, and, in some cases, also improves the vector’s efficiency as a biological transmitter by increasing opportunities for the acquisition and transmission of pathogens [85]. Since interrupted feeding does occur in ticks, the impact of such interrupted feeding on the duration of attachment required for pathogen transmission is relevant. Furthermore, the occurrence of intrastadial pathogen transmission is also relevant, which has been suspected for some time [86].

Shih and Spielman tested the effect of interrupted feeding on the dynamics of Lyme disease spirochetes transmission by infected *I. scapularis* nymphal ticks that reattached after feeding for various time periods on noninfected mice [31]. Their results showed that, after an initial feeding period of 24 h (on the initial host), 83% and 100% of mice (serving as the second host) acquired the infection after only 24 and 36 h, respectively, of tick attachment. The attachment duration for effective spirochete transmission was shortened by half in the interrupted feeding tick group; in the single-feeding tick group, 48 h were required for infection. All the second host mice became infected within 24 h when ticks were allowed to prefeed for 48 h on the initial host. Additionally, the results showed that uninfected *I. scapularis* nymphal ticks acquired *B. burgdorferi* from infected mice, and these ticks were able to transmit the infection to uninfected mice in less than 24 h [31].

Saraiva and collaborators demonstrated the activation and transmission of *R. rickettsii* by *A. aureolatum* nymphs and adults who experienced an interruption in feeding on naïve rabbits and guinea pigs. The results showed that an initial feeding phase of 48 h for male ticks on rabbits, followed by an immediate transfer to the second host, induced infection in one of two guinea pigs after only 10 min of attachment. Moreover, all animals (2/2) became infected after 20 min of attachment to the second host. The minimum time necessary for previously unfed *A. aureolatum* ticks to transmit *R. rickettsii* was estimated to be 12 h [19]. This difference in time to transmission between unfed and prefed *A. aureolatum* ticks infesting guinea pigs may be related to the reactivation of *R. rickettsii* accompanied by ultrastructural and physiological changes [87].

More recently, Varloud et al. demonstrated the ability of protozoan-infected prefed male *D. reticulatus* ticks to transmit *B. canis* to dogs within 8 h after infestation. Infected *D. reticulatus* ticks were permitted to prefeed on the initial hosts (sheep or dog) for a period of 88 h. Ticks were then detached manually and transferred to the second host (dogs) for just 8 h prior to tick removal. While the minimum transmission time necessary for unfed *D. reticulatus* ticks to transmit *B. canis* to dogs was expected to be 48 h based on microscopic observations of pathogen development and sporogony [88], the results of this study showed that 50% (3/6) of dogs fed on for 8 h by prefed ticks acquired babesiosis, which was confirmed with positive blood smears. Similarly, in rodents, laboratory data showed that a minimum of 36 h was required to induce infection in hamsters infested with *Babesia-microti*-infected *I. scapularis* ticks [20]. Interrupted feeding reduced the minimum transmission time necessary for the ticks to transmit *B. microti* from 36–48 h to just 8 h.

The cited examples illustrate faster transmission times for both bacterial and protozoal pathogens by partially fed (interrupted) ticks. However, no experimental information was found on the impact of interrupted feeding on the time to transmission of tick-borne viruses or nematodes. Typically, the transmission time for tick-borne viruses is short; for example, transmission of Powassan virus by *I. scapularis* nymphs can reportedly occur after only 15 min [15].

Human factors may also play a role in facilitating interrupted feeding in ticks and the potential for an increased transmission speed and risk of infection in humans and animals. Interrupted feeding and increased risk might occur in animal slaughter and game hunting situations, where partially fed ticks would be likely to leave their dead hosts as the body cools [66]. The consequences of controlling rodents using rodenticidal baits, a practice performed worldwide, especially on farmsteads, against rodents, which are well-known reservoirs of many tick-borne pathogens (e.g., *Borrelia burgdorferi, Francisella tularenis, Rickettsia* spp., and *Bartonella* spp.), deserve to be further investigated. Often, these rodents are infested with immature ticks. The presence of partially engorged, infected ticks in such situations could significantly increase the risk of tick-borne pathogen transmission to humans and pets.

## 5. Conclusions

Although interrupted feeding is known to frequently occur among flying, blood-sucking insects, particularly tabanids, interrupted feeding and mixed blood meals in ticks remain controversial topics. However, evidence shows that when ticks spontaneously detach themselves or are mechanically detached from an initial host, they are then able to survive for a certain length of time by switching their metabolism until they acquire a new host, on which they are able to reattach and complete engorgement. Interrupted feeding has been observed and documented in ticks under controlled conditions, which make such observations possible. Under natural conditions, the outcomes of interrupted feeding in ticks are more difficult to assess. However, indirect evidence, such as the detection of mixed host blood in questing nymphs and, unexpectedly, *B. burgdorferi*-infected host-seeking larvae, suggests that interrupted feeding occurs naturally. Taken together, these results suggest that interrupted feeding in ixodid ticks does indeed occur and that it may be a contributing factor to increasing the transmission risk of tick-borne pathogens to humans and animals.

Experimental studies on the transmission dynamics of tick-borne pathogens in cases of interrupted feeding will be required to generate accurate knowledge about, and reliable prevention strategies against, human and companion animal tick-borne diseases.

## Figures and Tables

**Table 1:** Transmission times for selected tick-borne pathogens under uninterrupted and interrupted tick feeding conditions.

Tick-Borne Pathogens	Tick Species	Stages	Transmission Time		References
			Uninterrupted Conditions	Interrupted Feeding	
*Borrelia burgdorferi* sensu stricto	*Ixodes scapularis*	Nymphs	36 h	24 h	[31]
*Rickettsia rickettsii*	*Amblyomma aureolatum*	Adults	12 h	10 min	[19]
*Babesia canis*	*Dermacentor reticulatus*	Adults	˃48 h	8 h	[32]

**Table 2:** Summary of publications reporting infection rates of *Borrelia burgdorferi* sensu lato in unfed, field-collected *Ixodes scapularis* and *Ixodes ricinus* larvae.

Country	Species	Year of Collection	Infection Rate		References
			%	n/N	
The Netherlands	*I. ricinus*	1993	17.6	21/84	[56]
The Netherlands	*I. ricinus*	1995	5	3/57	[57]
Germany	*I. ricinus*	2004	3.3	2/60	[58]
Italy	*I. ricinus*	2005	1.2	7/571	[59]
USA	*I. scapularis*	2008	/	1	[60]
Romania	*I. ricinus*	2010	0.4	2/545	[61]
Germany	*I. ricinus*	2010	25.8	8/31	[62]
The Netherlands	*I. ricinus*	2014	0.6	9/1456	[38]

**Table 3:** Evidence for interrupted feeding behavior in ixodid ticks.

Tick species	Parameters						References
	Tick Detachment	Tick Survival after Detachment	Tick Migration to a New Host	Tick Reattachment/Resumption of Blood Meal	Mixed Blood Meal	Spirochete-Infected Larvae	
*Ixodes ricinus*	/	/	/	/	Yes	Yes	[38,45,46,48,60,63]
*Ixodes scapularis*	Yes	Yes	Yes	Yes	Yes	/	[31,50,63,64]
*Ixodes persulcatus*	Yes	Yes	/	Yes	/	/	[30,37]
*Haemaphysalis spinigera*	/	/	/	Yes	/	/	[35]
*Hyalomma marginatum isaaci*	/	/	/	Yes	/	/	[36]
*Rhipicephalus appendiculatus*	Yes	Yes	/	Yes	/	/	[33,40]
*Rhipicephalus sanguineus*	Yes	Yes	Yes	Yes	/	/	[32,43]
*Dermacentor reticulatus*	/	/	Yes	Yes	/	/	[42,65]
*Dermacentor andersoni*	Yes	Yes	Yes	/	/	/	[40]
*Amblyomma americanum*	/	/	/	/	Yes	/	[50,51,52]
*Amblyomma aureolatum*	/	/	/	Yes	/	/	[66]
